# Risk factors for intracerebral hemorrhage in patients undergoing maintenance hemodialysis

**DOI:** 10.3389/fneur.2023.1111865

**Published:** 2023-03-22

**Authors:** Song Yu-Huan, Cai Guang-Yan, Xiao Yue-Fei

**Affiliations:** ^1^Department of Nephrology, Aerospace Center Hospital, Beijing, China; ^2^Department of Nephrology, State Key Laboratory of Kidney Diseases, Chinese PLA General Hospital, Chinese PLA Institute of Nephrology, National Clinical Research Center for Kidney Diseases, Beijing, China

**Keywords:** intracerebral hemorrhage, hemodialysis, parathyroid hormone, risk factor, secondary hyperparathyroidism

## Abstract

**Background:**

In patients undergoing hemodialysis, intracerebral hemorrhage (ICH) is the main cause of mortality among stroke subtypes. It is unclear whether, along with traditional cardiovascular risk factors, the risk factors unique to the uraemic environment, such as the abnormal metabolism of intact parathyroid hormone (iPTH), can contribute to the risk of ICH in these patients.

**Methods:**

This retrospective case–control study included 25 patients undergoing hemodialysis with ICH at a single center between 30 June 2015 and 10 October 2022. The controls were 95 patients undergoing maintenance hemodialysis treated at the same dialysis center in July 2020. We compared the characteristics of patients with ICH with those of the control group to identify factors that contributed to the development of ICH.

**Results:**

Intracerebral hemorrhage (ICH) was located in the basal ganglia (14/25), cerebellum (6/25), and brainstem (6/25) in 25 patients. A total of 17 patients died in the first 16 days due to neurological complications. Univariate analysis showed significant differences in systolic BP, diastolic BP, iPTH, and alkaline phosphatase between the two groups (*p* < 0.05). Multivariate logistic regression analysis showed that higher systolic BP (OR, 1.053; 95% CI, 1.018–1.090; *p* = 0.003) and higher iPTH (OR, 1.007; 95% CI, 1.003–1.012; *p* = 0.001) were associated with the onset of ICH. ICH was predicted by systolic BP and iPTH by receiver operating characteristic (ROC) curve analysis, with areas under the curve (AUCs) of 0.732 and 0.624, respectively. The optimal cutoffs for systolic BP and iPTH were 151.9 mmHg and 295.4 pg./ml, respectively. Restricted cubic spline showed that the shape of the association of iPTH with the risk of ICH was approximately J-shaped (P for non-linearity <0.05).

**Conclusion:**

Higher systolic BP and abnormal iPTH metabolism might be associated with ICH in patients undergoing hemodialysis. Comprehensive control of hypertension and iPTH may be a fundamental preventive strategy for ICH in these patients.

## Introduction

In patients undergoing dialysis, intracerebral hemorrhage (ICH) is the main cause of mortality among stroke subtypes (1). Therefore, it is important to formulate strategies that effectively minimize hemorrhage stroke risk in patients with advanced kidney disease. However, most studies of patients with end-stage renal disease (ESRD) have focused on ischemic stroke or all strokes combined, and fewer are focused on the presentation of symptoms, lesion location, and relative risks of ICH. Comorbid conditions, such as hypertension and diabetes, which are traditional risk factors for cerebral hemorrhage, are commonly observed in patients undergoing dialysis (2). Vascular calcification, predialysis hypertension, accumulation of vascular toxins, platelet disorder, and increased brain microbleeding are common features of the uraemic milieu that contribute to stroke risk (3). Chronic kidney disease–mineral-bone disorder (CKD-MBD) in patients undergoing hemodialysis may lead to accelerated vascular calcification (4). High serum calcium and phosphate levels are known risk factors for ICH in patients undergoing dialysis (5, 6).

One of the principal characteristics of CKD-MBD is the abnormal metabolism of iPTH. Patients may show either secondary hyperparathyroidism (SHPT) or low iPTH levels. Both of these internal secretion abnormalities are related to higher cardiovascular and all-cause mortality (7, 8). However, the association of iPTH and ICH is an uncertain area in the literature. Therefore, the purpose of this study was to investigate the risk factors for ICH in patients undergoing hemodialysis, including iPTH.

## Methods

The studies involving human participants were reviewed and approved by the Human Ethics Committee of Aerospace Center Hospital. The patients/participants provided their written informed consent to participate in this study. Written informed consent was obtained from the individual(s) and/or minor(s)’ legal guardian/next of kin for the publication of any potentially identifiable images or data included in this article.

### Entry criteria and definitions

This was a retrospective case–control study. The subjects were patients with cerebral hemorrhage who had been on hemodialysis (4 h, three times weekly) at Aerospace Center Hospital for more than 3 months. The study period was from 30 June 2015 to 10 October 2022. We selected patients with ICH using the electronic medical record system, which involved a continuous, broad search of all patients admitted to the emergency, neurological, neurosurgery, nephrology, or other relevant hospital wards for neurological indications that could be diagnosed as ICH. The diagnosis of ICH was based on computed tomography (CT) findings or magnetic resonance imaging and clinical signs, such as the sudden onset of disturbances in consciousness, speech, visual, or sensory functions. Patients with subarachnoid hemorrhage, traumatic brain injury, and hemorrhage after cerebral infarction stroke were excluded.

The control group consisted of all patients undergoing clinical maintenance hemodialysis with no clinical evidence of ICH and complete laboratory results who were admitted to Aerospace Center Hospital in July 2020. The data collection of patients with ICH was based on an outpatient routine blood test performed about 1 month before the onset of ICH, rather than the results obtained during the hospitalization period after the onset of ICH. The routine blood test of the control group was performed in July 2020. The routine blood samples in both groups were obtained before the second hemodialysis session of the week. Bicarbonate dialysis fluid containing 138 mEq/l sodium, 2.0 mEq/l potassium, and 1.5 mEq/l calcium was delivered during hemodialysis. If the blood calcium level of patients undergoing dialysis was above 2.5 mEq/l before dialysis, 1.25 mEq/l calcium was given. All patients undergoing hemodialysis received doses of 60 IU/kg low-molecular-weight heparins (LMWHs) to prevent clotting during hemodialysis, which could be adjusted at subsequent sessions when clinically indicated, by increments or decrements of 500 or 1,000 IU. LMWHs were administered intravenously as a single fixed bolus at the start of an HD session for up to 4 h. LMWHs were discontinued immediately when the patient was diagnosed with ICH or other bleeding events. Patients who had SHPT were administered a proper diet regimen, along with the administration of phosphate binders and the use of vitamin D to decrease the synthesis and secretion of iPTH. Patients who had undergone complete or partial parathyroid resection were excluded from both groups.

We analyzed the clinical features of patients with ICH, including demographics, comorbidities, laboratory values, and brain CT films at the onset of the disease. The corrected serum calcium values were calculated using the Payne equation (9). We compared the characteristics of ICH patients with those of the control group to identify factors that contributed to the development of ICH.

### Statistical analysis

We used SPSS version 22.0 (SPSS Inc., Chicago, IL, United States) and R version 4.2.1 (R Core Team) to carry out the statistical analysis. Categorical data were presented as counts or percentages, and continuous data were presented as mean ± SD or median (interquartile range). Comparisons between categorical data were fulfilled by the chi-square test, and continuous data were compared using the independent-samples t-test or variance analysis. To measure the relative risk of potential predictor variables, odds ratios (ORs) and 95% confidence intervals (95% CIs) were acquired by logistic regression analysis. Clinical parameters that were significant on univariate evaluation when *p*-values of ≤0.05 were appraised as potential predictor variables and evaluated by multivariate (stepwise forward) analysis. Receiver operating characteristic (ROC) curves were drawn and the area under the ROC curves (AUCs) were calculated to determine the predictive power of the variables for ICH. The cutoff points were determined based on the highest value for sensitivity added to the specificity judged by the ROC curve. Two-sided *p*-values of <0.05 were considered statistically significant. We performed restricted cubic splines with five knots at the 25, 35, 50, 65, and 95th centiles to flexibly model the relationship of predicted iPTH with ICH.

## Results

### The clinical characteristics of patients undergoing ICH dialysis

**Figure 1:**
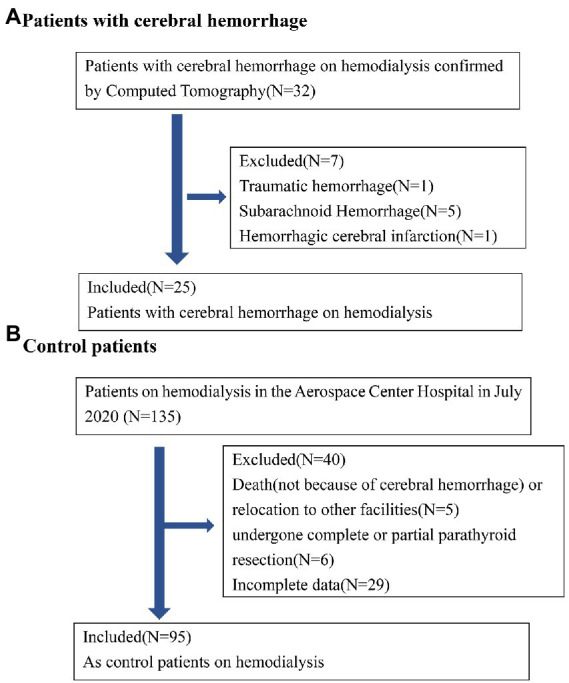
Patient flow diagram. **(A)** Hemodialysis with cerebral hemorrhage in Aerospace Center Hospital of China between July 1, 2015 and October 10, 2022; **(B)** control hemodialysis patients in Aerospace Center Hospital of China in July 2020.

A flow diagram of patients with ICH is shown in Figure 1. In total, 32 patients with cerebral hemorrhage on hemodialysis were confirmed by computed tomography. Five patients with subarachnoid hemorrhage, one patient with traumatic hemorrhage, and one patient with hemorrhagic cerebral infarction were excluded. Finally, 25 patients with ICH were included in this study. The demographic characteristics of 25 patients undergoing ICH dialysis (8 female and 17 male patients) are shown in the Supplementary FigureSupplementary Table. The ages of these 25 patients ranged from 31 to 92 years, with a mean age of 65.00 ± 13.19 years. The dialysis vintage was 43.56 ± 33.89 months (4–159). The mean systolic BP was 165.00 ± 19.41 mmHg. The mean diastolic BP was 81.96 ± 12.24 mmHg. Diabetes (64.0%) was the main cause in patients with hemorrhagic lesions causing ESRD.

As shown in the Supplementary FigureSupplementary Table, the signs of ICH in our study were a disturbed level of consciousness (11/25), limb dysfunction (7/25), headache (7/25), vomiting (6/25), impaired speech (5/25), or unsteady gait (1/25). All 25 patients had one or more symptoms. The Supplementary FigureSupplementary Figure shows the CT characteristics, hemorrhagic areas, and distribution of the hematoma in 25 patients with ICH. ICH was located in the basal ganglia (14/25), cerebellum (6/25), brainstem (6/25), thalamus (4/25), cerebral lobes (3/25), and ventricle (2/25). In patients undergoing hemodialysis, the ICH was severe in terms of hematoma size and clinical outcome. In the Supplementary FigureSupplementary Table, the size of the hemorrhagic lesion was usually large and the incidence of rupture into the ventricles was high. A total of 17 patients died in the first 16 days because of neurological complications. Only seven of the surviving patients recovered fully with no lasting sequelae.

Table 1 shows the clinical data of cases with ICH and controls. The predialysis systolic BP was 165.0 ± 19.41 mmHg in patients with ICH, which was significantly higher than that in the control group (145.17 ± 21.73 mmHg) (*p* < 0.001). The diastolic BP was 81.96 ± 12.24 mmHg in patients with ICH, which was also significantly higher than that in the control group (75.26 ± 11.84 mmHg) (*p* = 0.014). The serum iPTH was 266.00 (134.85–579.14) pg./ml in patients with ICH, which was significantly higher than that in the control group of 144.10 (198.10–265.70) pg./ml (*p* < 0.001). The serum alkaline phosphatase in the ICH group was 72.00(55.0–108.00) mmol/L, which was higher than that in the control group of 63.40 (53.10–82.00)mmol/L (*p* = 0.05). The nutritional status (hemoglobin and albumin) showed no significant differences. No differences were found in age, dialysis vintage, Kt/V, phosphorus, creatinine, triglycerides, or cholesterol (*p* > 0.05).

**Table 1:** Baseline characteristics of the study subjects.

Variable	Intracerebral hemorrhage group	Control group	*p*
Demographics			
Male(%)	17(68.0%)	55(57.9%)	0.199
Age, years	64.55 ± 13.56	60.85 ± 12.51	0.147
Dialysis vintage, months	43.85 ± 34.84	43.80 ± 39.28	0.978
Systolic BP, mmHg	163.82 ± 20.41	145.17 ± 21.73	**<0.001**
Diastolic BP, mmHg	81.96 ± 12.24	75.26 ± 11.84	**0.014**
Dialysis catheter	13(52.0%)	46(48.4%)	0.801
interdialytic weight gain	2.52 ± 0.70	2.47 ± 0.77	0.799
Comorbidities, *n*(%)			
Diabetic mellitus	16(64.0%)	47(49.5%)	0.462
Previous CVD	15(60.0%)	45(47.3%)	0.261
Smoking	5(20.0%)	14(14.7%)	0.521
Medication history, *n*(%)			
Dihydropyridine CCBs	15(60.0%)	60(63.2%)	0.772
ACEI/ARB	9(36.0%)	33(34.8%)	0.906
*β*-receptor blocker	6(24.0%)	15(15.8%)	0.336
Warfarln	4(16.0%)	5(5.4%)	0.070
Aspirin	6(24.0%)	19(20.0%)	0.661
Clopidogrel bisulfate	5(20.0%)	15(15.8%)	0.615
Laboratory values			
Serum creatinine, umol/L	787.25 ± 262.53	788.28 ± 281.38	0.656
Serum uric acid, umol/L	368.23 ± 83.39	411.10 ± 91.28	**0.036**
Serum calcium, mmol/L	2.31 ± 0.23	2.28 ± 0.19	0.590
Serum phosphorus, mmol/L	1.88 ± 0.64	1.94 ± 0.48	0.618
Alkaline phosphatase, U/L	72.00(55.0–108.00)	63.40(53.10–82.00)	**0.050**
IPTH, pg./ml	266.00(134.85–579.14)	144.10(198.10–265.70)	**<0.001**
Hemoglobin, g/L	112.91 ± 15.69	109.60 ± 11.86	0.579
Albumin, g/L	37.34 ± 3.96	36.67 ± 3.19	0.389
Triglyceride, mmol/L	1.83 ± 0.94	1.82 ± 1.17	0.979
Cholesterol, mmol/L	3.65 ± 0.87	3.68 ± 1.15	0.889
Kt/V	1.22 ± 0.13	1.21 ± 0.22	0.252
High density lipoprotein, mmol/L	0.87 ± 0.35	1.03 ± 0.31	0.162

BP, blood pressure. CCB, calcium channel blocker; ACEI/ARB, angiotensin-converting enzyme inhibitors/angiotensin receptor blocker; CVD, cardiovascular disease; IPTH, Intact parathyroid hormone. Kt/V (where *K* is the dialyzer clearance of urea, *t* is the dialysis time, and *V* is the volume of distribution of urea). The meaning of the bold values is: *p* ≤ 0.05.

In addition, we compared the effects of sex, comorbidities, and medication history on the incidence of ICH. No difference existed in sex, comorbidity of diabetes, previous cardiovascular disease, smoking, hypotensive drugs, anticoagulation drugs, or the use of dialysis catheters between patients with ICH and the control group (*p* > 0.05).

The results of the multivariate logistic regression analysis of all the participants, which aimed to identify factors associated with ICH, are shown in Table 2. After adjustment for age, sex, and diastolic blood pressure, the systolic BP and iPTH were significantly associated with a higher risk of ICH (model 1). After additional adjustments for serum alkaline phosphatase (model 2) and antiplatelets (model 3, aspirin and clopidogrel are combined as one confounder as antiplatelets), the associations remained significant for ICH.

**Table 2:** Multivariate logistic regression for independent variables potentially related to the incidence of ICH.

Variable	Model 1		Model 2		Model 3	
	OR(95% CI)	*p*	OR(95% CI)	*p*	OR(95% CI)	*p*
Age, years	1.045(0.996–1.097)	0.075	1.048(0.997–1.101)	0.066	1.046(0.996–1.100)	0.075
Sex	0.563(0.169–1.872)	0.349	0.648(0.189–2.225)	0.491	0.649(0.189–2.229)	0.492
IPTH, pg./ml	1.006(1.002–1.010)	0.003	1.005(1.001–1.009)	0.008	1.005(1.001–1.009)	0.008
Systolic BP, mmHg	1.048(1.014–1.083)	0.005	1.052(1.016–1.089)	0.004	1.052(1.016–1.089)	0.004
Diastolic BP, mmHg	1.002(0.955–1.051)	0.934	0.999(0.952–1.049)	0.976	0.999(0.951–1.049)	0.958
ALP, mmol/L			0.214(0.030–1.530)	0.125	0.217(0.030–1.568)	0.130
Antiplatelets					1.310(0.347–4.951)	0.690

Model 1, adjusted for age, sex, systolic and diastolic blood pressure, and iPTH; Model 2, adjusted for age, sex, systolic and diastolic blood pressure, iPTH, and ALP. Model 3, adjusted for age, sex, systolic and diastolic blood pressure, iPTH, ALP, and antiplatelets. Aspirin and clopidogrel are combined as one confounder as antiplatelets. OR = odds ratios; 95% CI = 95% confidence interval; BP, blood pressure; IPTH, Intact parathyroid hormone; ALP, alkaline phosphatase.

**Figure 2:**
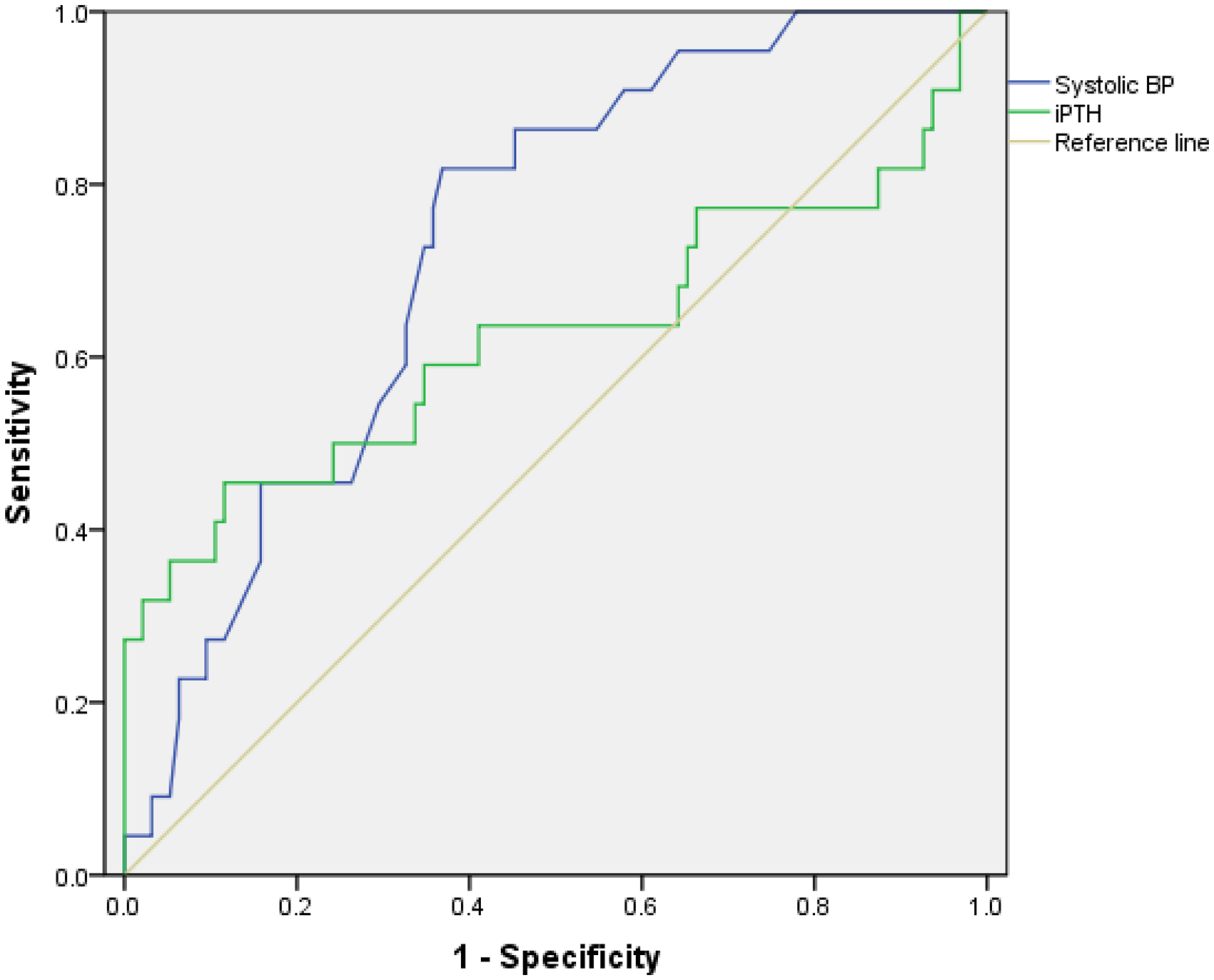
Receiver operating characteristic (ROC) curves of systolic BP and iPTH associated with ICH in patients undergoing maintenance hemodialysis.

ROC curves were constructed to compare the ability of systolic BP and iPTH to predict ICH (Figure 2). The results showed that ICH was predicted by systolic BP and iPTH, with AUCs of 0.732 and 0.624, respectively. The optimal cutoffs for systolic BP and iPTH were 151.9 mmHg and 295.4 pg./ml, respectively, according to the ROC curves.

**Figure 3:**
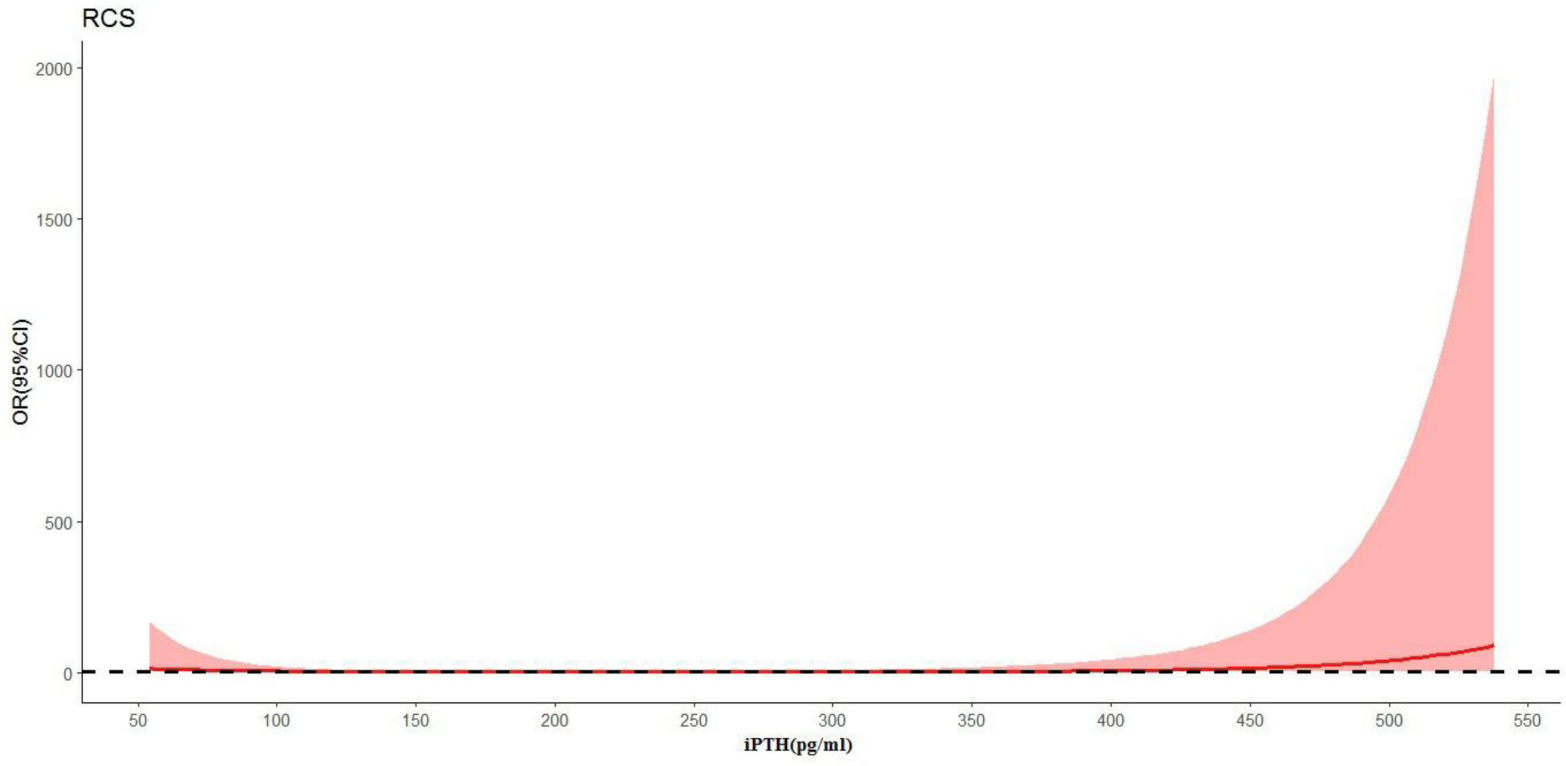
The relationship between the continuous change in iPTH and ICH (*p* = 0.024) using restricted cubic splines (RCS).

In Figure 3, the restricted cubic spline regression analysis shows that the nonlinear relationship between iPTH and ICH was J-shaped. The risk of ICH was relatively flat until iPTH reached approximately 295 pg./ml, and then the risk started to increase rapidly. The risk of ICH also increased when iPTH fell below 100 pg./ml.

## Discussion

In this study, the clinical outcomes and risk factors of ICH were analyzed in 25 patients undergoing maintenance hemodialysis. Outcomes after stroke in patients undergoing hemodialysis are worse than in general populations (10). In our study, about 60% of the ICH group died within 16 days of the onset of ICH. The risk of hemorrhagic stroke is high in patients undergoing hemodialysis (11).

The high mortality rate associated with ICH among patients undergoing hemodialysis is related to the large size of the hemorrhagic lesion and the high incidence of rupture into the ventricles (12). The use of anticoagulation to maintain dialysis circuits during hemodialysis may promote the enlargement of cerebral lesions (13). LMWHs have been suggested as providing well-tolerated, efficient, convenient, and possibly more cost-effective anticoagulation for hemodialysis than unfractionated heparins (UFHs) (14). Despite widespread use, there is little standardization of LMWHs dosing protocols (15). Anti-Xa activity measurement can be used to adjust the dosage of LMWHs and predict the anticoagulant effect during hemodialysis (16). As an anticoagulant in the extracorporeal circuit, sodium citrate has been widely used in continuous renal replacement therapy (17). The study showed that regional citrate anticoagulation combined with sequential anticoagulation and standard calcium-containing dialysate in intermittent hemodialysis treatment is safe and effective (18). The choice of anticoagulant therapy in patients undergoing hemodialysis should be determined by patient characteristics, local expertise, and ease of monitoring (19).

Hypertension is viewed as one of the predominant risk factors for ICH in patients undergoing hemodialysis (20). In this study, we found that the risk of ICH proportionally increased with a higher systolic BP at the start of hemodialysis. The optimal cutoff for predialysis systolic BP to predict ICH was 151.9 mmHg in this study. Systolic BP less than 151.9 mmHg in patients undergoing hemodialysis might be associated with a low risk of ICH incidence. Hypertension in patients undergoing dialysis often results from extracellular volume expansion, incrementally sympathetic activity, and vascular stiffness (21). Vascular stiffness reduces systolic compliance and diastolic flinch acting to increase systolic BP while simultaneously lowering BP (22, 23). One of the most important preventive strategies in the prevention of ICH is the treatment of hypertension (24). Hypervolemia is a major cause of hypertension in the population undergoing dialysis (25). Volume overload is a key modifiable contributor to hypertension and cardiovascular disease in the population undergoing hemodialysis (26). Initiation and the dose increase of antihypertensive medications therapy are proven to be beneficial only after the blood pressure remains reactionless to volume management methods (27).

CKD-MBD is characterized by abnormal mineral metabolism, bone turnover, and vascular calcification and is a prominent feature of CKD (28). Markers of CKD-MBD, such as increased serum iPTH, have been proposed as “emerging” stroke risk factors in patients undergoing dialysis (13). Current CKD-MBD guidelines recommend a wide range of serum PTH targets (2–9 times the upper limit of normal of the intact PTH assay) (29, 30). An increase in circulating iPTH is associated with a higher risk of brain hemorrhage (31). One large Japanese study including 65,849 patients undergoing hemodialysis detected that high iPTH levels as a risk factor for ICH in patients undergoing hemodialysis (32). Elevated iPTH may increase the risk for ICH through the following pathogenesis: higher serum iPTH concentrations have been associated with increased blood pressure and vascular contractility, which may eventually lead to hypertrophy, apoptosis, and fibrosis of the vascular smooth muscle (33). Elevated iPTH may also predispose to vascular calcification (34). It can stimulate cytokine release from vascular smooth muscle cells and lymphocytes and thus affect the cardiovascular system *via* pro-inflammatory effects (35). IPTH is linked to the renin–angiotensin–aldosterone system (RAAS) as a probable mechanism of hypertension and ICH (36, 37). IPTH levels are a risk factor for hypertension *via* vessel calcification so hypertension potentially remains the main direct risk factor for ICH. In hypertensive patients with hyperparathyroidism, parathyroidectomy has been associated not only with greater decreases in their blood pressure but also with decreased use of antihypertensive drugs (38–40). In addition, we also found that a lower level of iPTH (<100 pg./ml) increased the risk of ICH in patients undergoing hemodialysis. A low level of iPTH is a manifestation of the malnutrition–inflammation complex syndrome (41). Age, comorbid diabetes, and hypoalbuminaemia have been associated with low iPTH levels in patients undergoing dialysis. All of these are risk factors for cerebrovascular events (42). Rational iPTH control is thus significant (41). Higher serum phosphate levels are also likely to increase the risk of hemorrhagic stroke (5, 43).

In addition, univariate analysis in this study showed that the level of alkaline phosphatase is higher in the ICH group. This is consistent with literature reports. Kitamura found that elevated serum alkaline phosphatase levels are an important risk factor for brain hemorrhage in patients receiving maintenance hemodialysis (44). Alkaline phosphatase is a membrane-bound enzyme that catalyzes the conversion of pyrophosphate into inorganic phosphate and is used as a marker of high bone turnover in patients with CKD (45). High alkaline phosphatase levels also might serve as a predictor for poor functional outcomes after ICH onset (46). In our analysis, patients with high systolic BP and poorly managed chronic kidney disease/mineral bone disease have an increased risk of hemorrhagic stroke. These findings suggested that regular monitoring of iPTH and alkaline phosphatase levels may help in improving the early identification of the population at higher ICH risk.

The strengths of our study include the use of case–control study design to evaluate the associations of systolic BP and serum iPTH levels with incident ICH in patients undergoing hemodialysis. We identified high predialysis systolic blood pressure and high iPTH levels as risk factors for ICH in patients undergoing hemodialysis. This confirms former investigations and encourages to do regular checks of iPTH levels in patients undergoing hemodialysis.

Although the current results are instructive, this report has several limitations. First, because of the low incidence rate of ICH among patients undergoing dialysis, for example, it was approximately 0.6% in a large Taiwanese hemodialysis population (47), so we chose a retrospective case–control design in this study. Second, only patients with ICH undergoing dialysis who were admitted to the hospital were included. One stroke project found that up to 46% of patients with stroke emergencies were never admitted to the hospital (48). Accordingly, our assessment of hemorrhage stroke risk factors may be incomplete. Third, findings regarding hypertension as a risk factor for ICH are not original, even in end-stage kidney disease. It also remains unclear whether there is an independent correlation between iPTH and ICH or whether the observed findings are rather driven by hypertension in the ICH group. This should be added to the limitations of the study. In addition, potential bias due to the small sample size and corresponding low statistical power, and the small sample size that might limit the performance of the multivariate statistical analyses should be included in the limitations of the study.

In conclusion, ICH in patients undergoing hemodialysis is associated with significant mortality. Inadequate control of hypertension and iPTH remains two significant risk factors. These results indicate that the importance of the control of blood pressure and PTH among patients undergoing hemodialysis may show great potential as a way to reduce the incidence of ICH.

## Data availability statement

The raw data supporting the conclusions of this article will be made available by the authors, without undue reservation.

## Ethics statement

The studies involving human participants were reviewed and approved by the Human Ethics Committee of Aerospace Center Hospital. The patients/participants provided their written informed consent to participate in this study. Written informed consent was obtained from the individual(s) and/or minor(s)' legal guardian/next of kin for the publication of any potentially identifiable images or data included in this article.

## Author contributions

SY-H, CG-Y, and XY-F conceived and designed the study. SY-H and CG-Y participated in the literature search, data analysis, and interpretation. SY-H drafted the manuscript. CG-Y revised the final manuscript. All authors contributed to the design of the study and the interpretation of the data, read and approved the final manuscript.

## Funding

This study received financial support from the Scientific Research Fund of Aerospace Center Hospital (YN202209) and the Natural Science Foundation of China (NSFC) (82170686).

## Conflict of interest

The authors declare that the research was conducted in the absence of any commercial or financial relationships that could be construed as a potential conflict of interest.

## Publisher’s note

All claims expressed in this article are solely those of the authors and do not necessarily represent those of their affiliated organizations, or those of the publisher, the editors and the reviewers. Any product that may be evaluated in this article, or claim that may be made by its manufacturer, is not guaranteed or endorsed by the publisher.

## Supplementary material

The Supplementary material for this article can be found online at: https://www.frontiersin.org/articles/10.3389/fneur.2023.1111865/full#supplementary-material

## Abbreviations

ICH: intracerebral hemorrhage
iPTH: intact parathyroid hormone
SHPT: secondary hyperparathyroidism
CKD-MBD: chronic kidney disease–mineral-bone disorder
ORs: odds ratios
95% CIs: 95% confidence intervals
ESRD: end-stage renal disease
RAAS: renin–angiotensin–aldosterone system
SUA: serum uric acid
ROC: receiver operating characteristic
AUC: area under the ROC curve

## References

[1] Wakasugi M Matsuo K Kazama JJ Narita I. Higher mortality due to intracerebral hemorrhage in dialysis patients: a comparison with the general population in Japan. Ther Apher Dial. (2015) 19:45–9. doi: 10.1111/1744-9987.12192, PMID: 25196294

[2] Chen Y Zhan X Zhao Q Wei X Xiao J Yan C. Serum lipoprotein(a) and risk of hemorrhagic stroke among incident peritoneal dialysis patients: a large study from a single center in China. Ren Fail. (2019) 41:800–7. doi: 10.1080/0886022X.2019.1659151, PMID: 31498021 PMC6746282

[3] Sozio SM Coresh J Jaar BG Fink NE Plantinga LC Armstrong PA. Inflammatory markers and risk of cerebrovascular events in patients initiating dialysis. Clin J Am Soc Nephrol. (2011) 6:1292–300. doi: 10.2215/CJN.08350910, PMID: 21551022 PMC3109924

[4] Jono S McKee MD Murry CE Shioi A Nishizawa Y Mori K. Phosphate regulation of vascular smooth muscle cell calcification. Circ Res. (2000) 87:E10–7. 11009570 10.1161/01.res.87.7.e10

[5] Yamada S Tsuruya K Taniguchi M Tokumoto M Fujisaki K Hirakata H. Association between serum phosphate levels and stroke risk in patients undergoing hemodialysis: the Q-cohort study. Stroke. (2016) 47:2189–96. doi: 10.1161/STROKEAHA.116.013195, PMID: 27507862

[6] Kitamura M Tateishi Y Sato S Kitamura S Ota Y Muta K. Association between serum calcium levels and prognosis, hematoma volume, and onset of cerebral hemorrhage in patients undergoing hemodialysis. BMC Nephrol. (2019) 20:210. doi: 10.1186/s12882-019-1400-4, PMID: 31174486 PMC6555959

[7] Muppidi V Meegada SR Rehman A. Secondary hyperparathyroidism. StatPearls [internet]. Treasure Island, FL: StatPearls Publishing (2022). 32491754

[8] Shiizaki K Sumikado S Akizawa T. Low PTH level and adynamic bone disease in dialysis patients. Clin Calcium. (2004) 14:16–20. 15576948

[9] Payne RB Little AJ Williams RB Milner JR. Interpretation of serum calcium in patients with abnormal serum proteins. Br Med J. (1973) 4:643–6. doi: 10.1136/bmj.4.5893.643, PMID: 4758544 PMC1587636

[10] Power A Chan K Singh SK Taube D Duncan N. Appraising stroke risk in maintenance hemodialysis patients: a large single-center cohort study. Am J Kidney Dis. (2012) 59:249–57. doi: 10.1053/j.ajkd.2011.07.016. Epub 2011 Sep 23, PMID: 21944665

[11] Onoyama K Kumagai H Miishima T Tsuruda H Tomooka S Motomura K. Incidence of strokes and its prognosis in patients on maintenance hemodialysis. Jpn Heart J. (1986) 27:685–91. doi: 10.1536/ihj.27.685 3820579

[12] Kawamura M Fijimoto S Hisanaga S Yamamoto Y Eto T. Incidence, outcome, and risk factors of cerebrovascular events in patients undergoing maintenance hemodialysis. Am J Kidney Dis. (1998) 31:991–6. doi: 10.1053/ajkd.1998.v31.pm9631844, PMID: 9631844

[13] Herrington W Haynes R Staplin N Emberson J Baigent C Landray M. Evidence for the prevention and treatment of stroke in dialysis patients. Semin Dial. (2015) 28:35–47. doi: 10.1111/sdi.12281, PMID: 25040468 PMC4320775

[14] Sabry A Taha M Nada M Al Fawzan F Alsaran K. Anticoagulation therapy during haemodialysis: a comparative study between two heparin regimens. Blood Coagul Fibrinolysis. (2009) 20:57–62. doi: 10.1097/MBC.0b013e32831bec0f. PMID: 20523166, PMID: 20523166

[15] Claudel SE Miles LA Murea M. Anticoagulation in hemodialysis: a narrative review. Semin Dial. (2021) 34:103–15. doi: 10.1111/sdi.12932. Epub 2020 Nov 1, PMID: 33135208

[16] Tao M Zheng D Liang X Ye M Liu Y Li Y. Evaluation of the anticoagulant effect of low-molecular-weight heparins based on the anti-Xa level during haemodialysis. Nephrology (Carlton). (2020) 25:723–9. doi: 10.1111/nep.13697, PMID: 31999031

[17] Zarbock A Küllmar M Kindgen-Milles D Wempe C Gerss J Brandenburger T. Effect of regional citrate anticoagulation vs systemic heparin anticoagulation during continuous kidney replacement therapy on dialysis filter life span and mortality among critically ill patients with acute kidney injury: a randomized clinical trial. JAMA. (2020) 324:1629–39. doi: 10.1001/jama.2020.18618, PMID: 33095849 PMC7585036

[18] Tang X Chen D Zhang L Fu P Chen Y Xiao Z. Application of regional citrate anticoagulation in patients at high risk of bleeding during intermittent hemodialysis: a prospective multicenter randomized controlled trial. J Zhejiang Univ Sci B. (2022) 23:931–42. doi: 10.1631/jzus.B2200082, PMID: 36379612 PMC9676090

[19] Legrand M Tolwani A. Anticoagulation strategies in continuous renal replacement therapy. Semin Dial. (2021) 34:416–22. doi: 10.1111/sdi.12959, PMID: 33684244

[20] Wang HH Hung SY Sung JM Hung KY Wang JD. Risk of stroke in long-term dialysis patients compared with the general population. Am J Kidney Dis. (2014) 63:604–11. doi: 10.1053/j.ajkd.2013.10.013, PMID: 24290244

[21] Hörl MP Hörl WH. Hemodialysis-associated hypertension: pathophysiology and therapy. Am J Kidney Dis. (2002) 39:227–44. doi: 10.1053/ajkd.2002.30542, PMID: 11840363

[22] Guérin AP Pannier B Métivier F Marchais SJ London GM. Assessment and significance of arterial stiffness in patients with chronic kidney disease. Curr Opin Nephrol Hypertens. (2008) 17:635–41. doi: 10.1097/MNH.0b013e32830dcd5c 19031658

[23] London GM. Arteriosclerosis and arterial calcifications in chronic kidney insufficiency. Nephrol Ther. (2005) 1:S351–4. 17373207

[24] Diener HC Hankey GJ. Primary and secondary prevention of ischemic stroke and cerebral hemorrhage: JACC focus seminar. J Am Coll Cardiol. (2020) 75:1804–18. doi: 10.1016/j.jacc.2019.12.072 32299593

[25] Ozkahya M. Pharmacological and non-pharmacological treatment of hypertension in dialysis patients. Kidney Int Suppl. (2011) 3:380–2. doi: 10.1038/kisup.2013.82 PMC4089642 25028643

[26] Flythe JE Bansal N. The relationship of volume overload and its control to hypertension in hemodialysis patients. Semin Dial. (2019) 32:500–6. doi: 10.1111/sdi.12838, PMID: 31564065 PMC6848760

[27] Georgianos PI Agarwal R. Blood pressure control in conventional hemodialysis. Semin Dial. (2018) 31:557–62. doi: 10.1111/sdi.12741, PMID: 30084190 PMC6218270

[28] Isakova T Nickolas TL Denburg M Yarlagadda S Weiner DE Gutiérrez OM. KDOQI US commentary on the 2017 KDIGO clinical practice guideline update for the diagnosis, evaluation, prevention, and treatment of chronic kidney disease-mineral and bone disorder (CKD-MBD). Am J Kidney Dis. (2017) 70:737–51. doi: 10.1053/j.ajkd.2017.07.019, PMID: 28941764

[29] de Francisco AL. Secondary hyperparathyroidism: review of the disease and its treatment. Clin Ther. (2004) 26:1976–93. doi: 10.1016/j.clinthera.2004.12.011 15823762

[30] Kritmetapak K Pongchaiyakul C. Parathyroid hormone measurement in chronic kidney disease: from basics to clinical implications. Int J Nephrol. (2019) 2019:1–9. doi: 10.1155/2019/5496710 PMC6766083 31637056

[31] Zhang Y Zhang DZ. Circulating parathyroid hormone and risk of hypertension: a meta-analysis. Clin Chim Acta. (2018) 482:40–5. doi: 10.1016/j.cca.2018.03.028, PMID: 29596813

[32] Tagawa M Hamano T Nishi H Tsuchida K Hanafusa N Fukatsu A. Mineral metabolism markers are associated with myocardial infarction and hemorrhagic stroke but not ischemic stroke in hemodialysis patients: a longitudinal study. PLoS One. (2014) 9:e114678. doi: 10.1371/journal.pone.0114678 25494334 PMC4262415

[33] Korada SK Zhao D Gottesman RF Guallar E Lutsey PL Alonso A. Parathyroid hormone and subclinical cerebrovascular disease: the atherosclerosis risk in communities brain magnetic resonance imaging study. J Stroke Cerebrovasc Dis. (2016) 25:883–93. doi: 10.1016/j.jstrokecerebrovasdis.2015.12.029, PMID: 26825350 PMC4799747

[34] Andersson P Rydberg E Willenheimer R. Primary hyperparathyroidism and heart disease – A review. Eur Heart J. (2004) 25:1776–87. doi: 10.1016/j.ehj.2004.07.010 15474692

[35] Folsom AR Alonso A Misialek JR Michos ED Selvin E Eckfeldt JH. Parathyroid hormone concentration and risk of cardiovascular diseases: the atherosclerosis risk in communities (ARIC) study. Am Heart J. (2014) 168:296–302. doi: 10.1016/j.ahj.2014.04.017, PMID: 25173540 PMC4150218

[36] Kono K Fujii H Watanabe K Goto S Nishi S. Relationship between parathyroid hormone and renin-angiotensin-aldosterone system in hemodialysis patients with secondary hyperparathyroidism. J Bone Miner Metab. (2021) 39:230–6. doi: 10.1007/s00774-020-01139-5, PMID: 32920706

[37] Yagi S Aihara K Kondo T Endo I Hotchi J Ise T. High serum parathyroid hormone and calcium are risk factors for hypertension in Japanese patients. Endocr J. (2014) 61:727–33. doi: 10.1507/endocrj.EJ14-0004, PMID: 24849536

[38] Fujii H. Association between parathyroid hormone and cardiovascular disease. Ther Apher Dial. (2018) 22:236–41. doi: 10.1111/1744-9987.12679 29707916

[39] Tomaschitz A Ritz E Pieske B Rus-Machan J Kienreich K Verheyen N. Aldosterone and parathyroid hormone interactions as mediators of metabolic and cardiovascular disease. Metabolism. (2014) 63:20–31. doi: 10.1016/j.metabol.2013.08.016, PMID: 24095631

[40] Tomaschitz A Pilz S Rus-Machan J Meinitzer A Brandenburg VM Scharnagl H. Interrelated aldosterone and parathyroid hormone mutually modify cardiovascular mortality risk. Int J Cardiol. (2015) 184:710–6. doi: 10.1016/j.ijcard.2015.03.062, PMID: 25777071

[41] Raj R Kadiyala A Patel C. Malnutrition-inflammation complex syndrome: a cause of low parathyroid hormone in patients with chronic kidney disease. Cureus. (2021) 13:e20324. doi: 10.7759/cureus.20324, PMID: 35028221 PMC8743024

[42] Yu Y Diao Z Wang Y Zhou P Ding R Liu W. Hemodialysis patients with low serum parathyroid hormone levels have a poorer prognosis than those with secondary hyperparathyroidism. Ther Adv Endocrinol Metab. (2020) 11:204201882095832. doi: 10.1177/2042018820958322 PMC7513009 33014329

[43] Block GA Klassen PS Lazarus JM Ofsthun N Lowrie EG Chertow GM. Mineral metabolism, mortality, and morbidity in maintenance hemodialysis. J Am Soc Nephrol. (2004) 15:2208–18. doi: 10.1097/01.ASN.0000133041.27682.A2 15284307

[44] Kitamura H Yamada S Hiyamuta H Yotsueda R Taniguchi M Tokumoto M. Serum alkaline phosphatase levels and increased risk of brain hemorrhage in hemodialysis patients: the Q-cohort study. J Atheroscler Thromb. (2022) 29:923–36. doi: 10.5551/jat.62885, PMID: 34108341 PMC9174090

[45] Fukagawa M Yokoyama K Koiwa F Taniguchi M Shoji T Kazama JJ. CKD-MBD guideline working group; Japanese Society for Dialysis Therapy: clinical practice guideline for the management of chronic kidney disease-mineral and bone disorder. Ther Apher Dial. (2013) 17:247–88. doi: 10.1111/1744-9987.12058 23735142

[46] Li S Wang W Zhang Q Wang Y Wang A Zhao X. Association between alkaline phosphatase and clinical outcomes in patients with spontaneous intracerebral hemorrhage. Front Neurol. (2021) 12:677696. doi: 10.3389/fneur.2021.677696, PMID: 34526953 PMC8435581

[47] Lin CY Chien CC Chen HA Su FM Wang JJ Wang CC. The impact of comorbidity on survival after hemorrhagic stroke among dialysis patients: a nationwide population-based study. BMC Nephrol. (2014) 15:186. doi: 10.1186/1471-2369-15-186 25427630 PMC4256891

[48] Rothwell PM Coull AJ Giles MF Howard SC Silver LE Bull LM. Change in stroke incidence, mortality, case-fatality, severity, and risk factors in Oxfordshire, UK from 1981 to 2004 (Oxford vascular study). Lancet. (2004) 363:1925–33. doi: 10.1016/S0140-6736(04)16405-2, PMID: 15194251

