# Supplementary material for: Risk factors for intracerebral hemorrhage in patients undergoing maintenance hemodialysis
Source: Front Neurol. 2023 Mar 22;14:1111865. doi: 10.3389/fneur.2023.1111865 (PMC10073690; doi:10.3389/fneur.2023.1111865)
Supplement: Supplementary file 1 [file Data_Sheet_1.docx]

Supplementary Table. Characteristics of 25 cases of intracerebral hemorrhage

| Case | Sex/ age | Primary renal disease | iPTH(pg/ml) | Vascular Access Type | Symptom o ICH | Location of ICH | Puncture drainage | Prognosis of ICH |
| --- | --- | --- | --- | --- | --- | --- | --- | --- |
| 1 | F/73 | Diabetes | 238.0 | Arteriovenous graft | Loss of consciousness | Cerebellum and brainstem | No | Died in 2 days |
| 2 | M/78 | Hypertension | 173.0 | Arteriovenous fistula | Limb weakness and influential speech | L basal ganglia | No | Died in 2 days |
| 3 | M/71 | Diabetes | 50.9 | Arteriovenous fistula | Limb weakness and influential speech | Ventricle | No | Died in 3 days |
| 4 | M/53 | Glomerulonephritis | 684.0 | Arteriovenous fistula | Headache and limb weakness | R basal ganglia | No | Died in 3 days |
| 5 | M/73 | Diabetes | 297.0 | Central venous catheter | Loss of consciousness and vomiting | Brainstem | No | Died in 16 days |
| 6 | M/64 | Hypertension | 69.0 | Arteriovenous fistula | Loss of consciousness | L basal ganglia | Yes | Died in 2 days |
| 7 | F/92 | Diabetes | 246.0 | Central venous catheter | Loss of consciousness and vomiting | R basal ganglia | No | Died in 15 hours |
| 8 | F/51 | Glomerulonephritis | 110.0 | Central venous catheter | Headache | R basal ganglia | No | Died in 14 days |
| 9 | F/60 | Hypertension | 213.0 | Arteriovenous fistula | Fatigue | R thalamus | No | Died in 4 days |
| 10 | F/81 | Diabetes | 156.0 | Central venous catheter | Limb weakness and vomiting | R thalamus | No | Alive |
| 11 | M/60 | Diabetes | 376.0 | Arteriovenous fistula | Headache and vomiting | Cerebellum and brainstem | Yes | Alive |
| 12 | M/59 | Diabetes | 401.0 | Arteriovenous fistula | Loss of consciousness | Brainstem | Yes | Died in 1 years |
| 13 | M/47 | Diabetes | 60.4 | Arteriovenous fistula | Loss of consciousness and vomiting | Cerebellum and brainstem | No | Died in 7 days |
| 14 | M/76 | Hypertension | 845.0 | Arteriovenous fistula | Loss of consciousness and vomiting | Cerebellum and brainstem | Yes | Died in 1 year |
| 15 | M/82 | Diabetes | 48.9 | Arteriovenous fistula | Fatigue | Ventricular | Yes | Alive |
| 16 | M/55 | Diabetes | 966.0 | Arteriovenous fistula | Unsteady walking with influential speech | L basal ganglia | No | Alive |
| 17 | M/56 | Diabetes | 266.0 | Arteriovenous fistula | Headache and limb weakness | L thalamus | No | Died in 1 year |
| 18 | F/54 | Diabetes | 167.0 | Arteriovenous fistula | Headache | R temporal lobe and basal ganglia | No | Died in 9 months |
| 19 | M/74 | Hypertension | 140.5 | Arteriovenous fistula | Loss of consciousness | R temporal lobe and basal ganglia | No | Died in 1 days |
| 20 | M/68 | Diabetes | 153.5 | Arteriovenous fistula | Loss of consciousness | R temporal lobe and basal ganglia | No | Died in 1 days |
| 21 | M/59 | Glomerulonephritis | 580.3 | Central venous catheter | Influential speech | L basal ganglia | No | Alive |
| 21 | M/59 | Glomerulonephritis | 580.3 | Central venous catheter | Influential speech | L basal ganglia | No | Alive |
| 22 | M/31 | Glomerulonephritis | 845.0 | Central venous catheter | Loss of consciousness | L basal ganglia | Yes | Died in 7 days |
| 23 | F/55 | Diabetes | 314.1 | Arteriovenous fistula | Limb weakness | L basal ganglia | No | Alive |
| 24 | M/77 | Diabetes | 113.7 | Arteriovenous fistula | Headache | L basal ganglia | No | Alive |
| 25 | F/73 | Glomerulonephritis | 785.0 | Arteriovenous fistula | Loss of consciousness | R basal ganglia and thalamus | No | Died in 7 days |

Abbreviations: ICH,intracerebral hemorrhage; M,male; F,female; L,left; R,right; iPTH, intact prathyroid hormone.


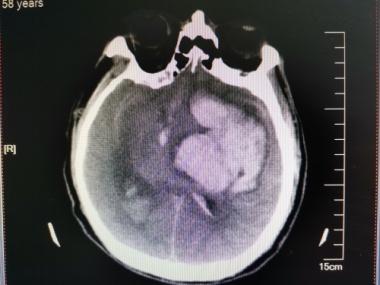


Case 1


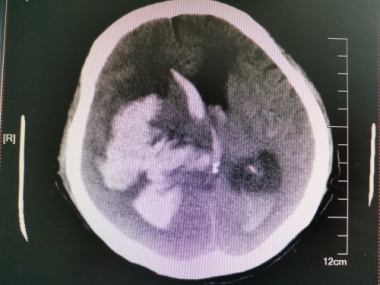


Case 4


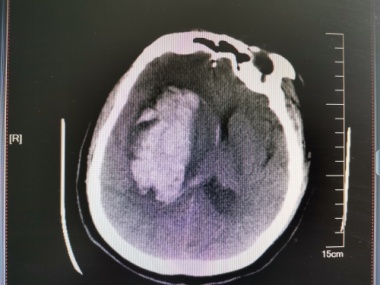


Case 7


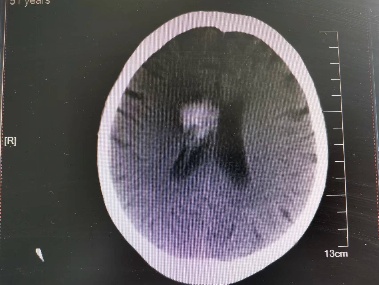


Case 10


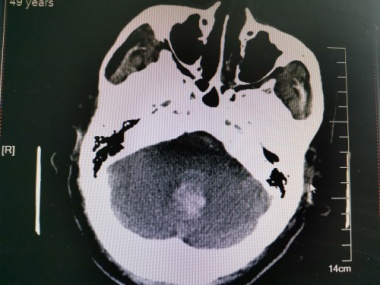


Case 13


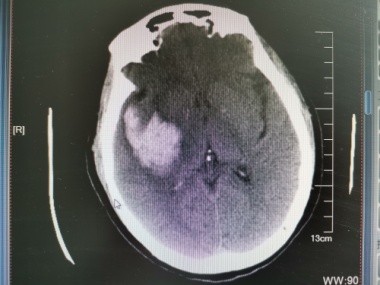


Case 16


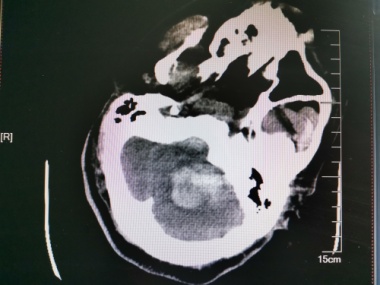


Case 2


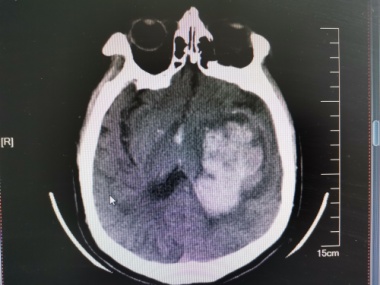


Case 5


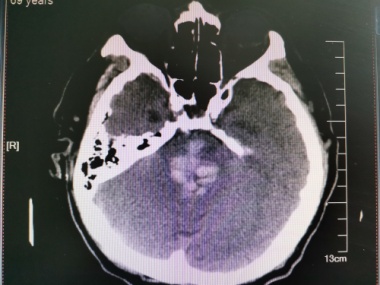


Case 8


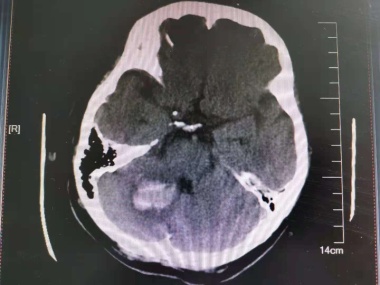


Case 11


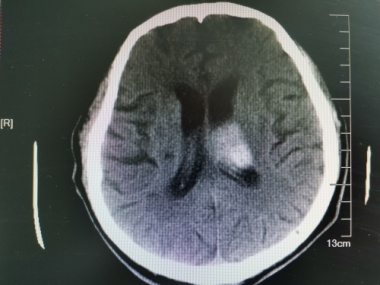


Case 14


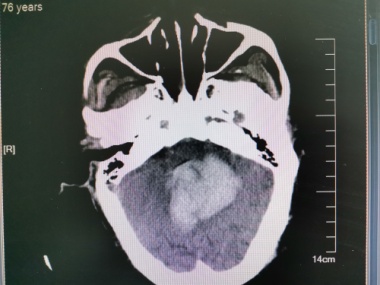


Case 17


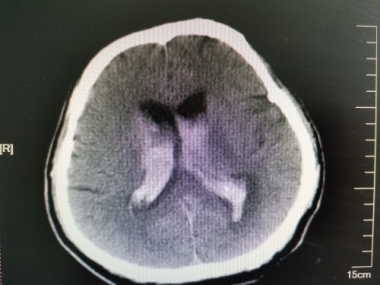


Case 3


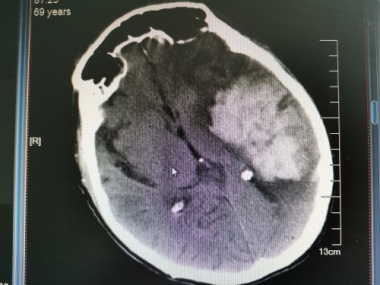


Case 6


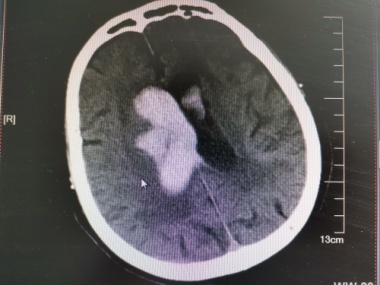


Case 9


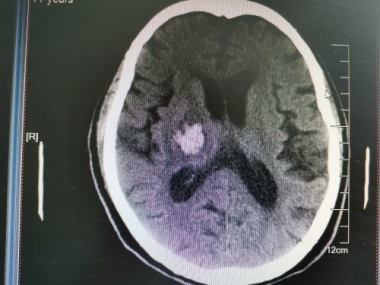


Case 12


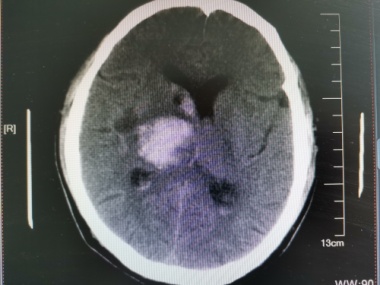


Case 15


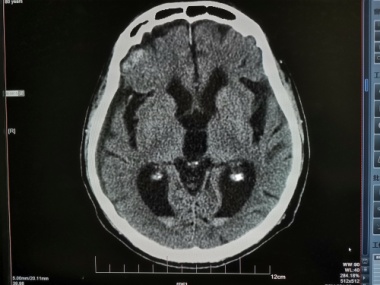


Case 18


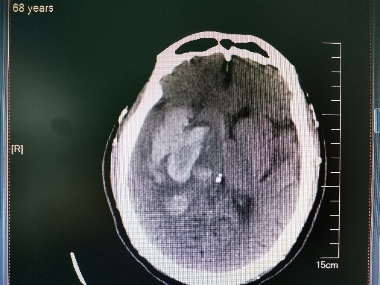


Case 19


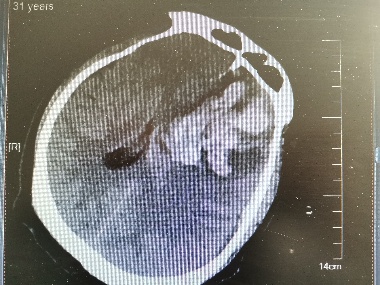


Case 22


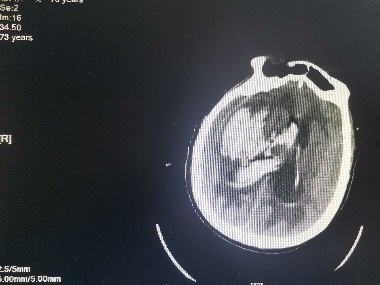


Case 25


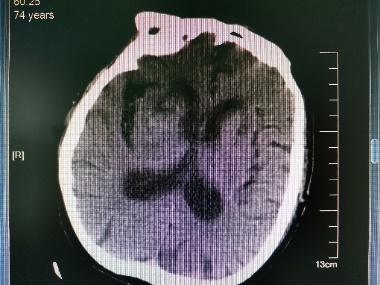
Case 20


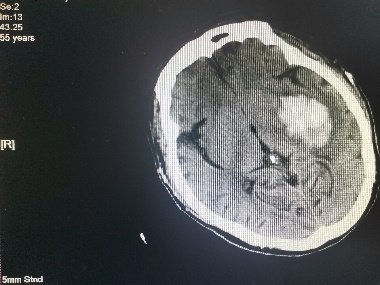


Case 23


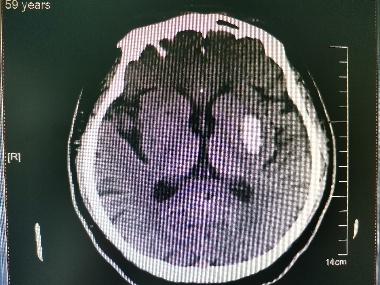


Case 21


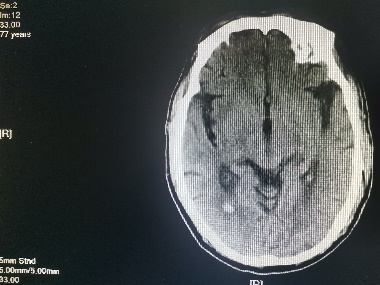


Case 24

Supplementary Figure. Computed tomographic or magnetic resonance imaging of intracranial hemorrhage in 25 hemodialysis patients
